# A Survival Association Study of 102 Polymorphisms Previously Associated with Survival Outcomes in Colorectal Cancer

**DOI:** 10.1155/2015/968743

**Published:** 2015-05-12

**Authors:** Sevtap Savas, Jingxiong Xu, Salem Werdyani, Konstantin Shestopaloff, Elizabeth Dicks, Jane Green, Patrick Parfrey, Roger Green, Wei Xu

**Affiliations:** ^1^Discipline of Genetics, Faculty of Medicine, Memorial University, St. John's, NL, Canada A1B 3V6; ^2^Discipline of Oncology, Faculty of Medicine, Memorial University, St. John's, NL, Canada A1B 3V6; ^3^Department of Biostatistics, Princess Margaret Hospital, Toronto, ON, Canada M5G 2M9; ^4^Dalla Lana School of Public Health, University of Toronto, Toronto, ON, Canada M5T 3M7; ^5^Clinical Epidemiology Unit, Faculty of Medicine, Memorial University, St. John's, NL, Canada A1B 3V6

## Abstract

Several published studies identified associations of a number of polymorphisms with a variety of survival outcomes in colorectal cancer. In this study, we aimed to explore 102 previously reported common genetic polymorphisms and their associations with overall survival (OS) and disease-free survival (DFS) in a colorectal cancer patient cohort from Newfoundland (*n* = 505). Genotypes were obtained using a genomewide SNP genotyping platform. For each polymorphism, the best possible genetic model was estimated for both overall survival and disease-free survival using a previously published approach. These SNPs were then analyzed under their genetic models by Cox regression method. Correction for multiple comparisons was performed by the False Discovery Rate (FDR) method. Univariate analysis results showed that *RRM1*-rs12806698, *IFNGR1*-rs1327474, *DDX20*-rs197412, and *PTGS2*-rs5275 polymorphisms were nominally associated with OS or DFS (*p* < 0.01). In stage-adjusted analysis, the nominal associations of *DDX20*-rs197412, *PTGS2*-rs5275, and *HSPA5*-rs391957 with DFS were detected. However, after FDR correction none of these polymorphisms remained significantly associated with the survival outcomes. We conclude that polymorphisms investigated in this study are not associated with OS or DFS in our colorectal cancer patient cohort.

## 1. Background

Colorectal cancer is one of the most frequently diagnosed malignancies. Advances in treatment and implementation of screening programs decrease its incidence and mortality rates; however, the current 5-year survival rate for colorectal cancer is considered only “relatively good” (62–65%) in North America [1, 2]. In other parts of the world, the survival rates for this disease are more disappointing (30–54%) [3].

Variability between the initial prognostic estimations and the actual outcomes observed in patients underlines the need to improve the prognostic estimations. Additional and possibly multiple prognostic predictors may help improve the prediction models by helping better discriminating the patients with different possible disease outcomes [4].

Polymorphic genetic variations, such as single nucleotide polymorphisms (SNPs), exist in large numbers in the human genome [5]. Genetic polymorphisms are candidate prognostic markers because of their variability among different individuals and their potential role as the biological modifiers of outcome risk. Similar to other cancer sites, in colorectal cancer several polymorphisms have been identified as being associated with a variety of survival outcomes.

The aim of this study was to investigate whether polymorphisms that were reported in the literature to be associated with survival outcomes were associated with select survival outcomes in our patient cohort. Specifically, we tested the associations of 102 SNPs from 73 genes and genetic loci with overall and disease-free survivals in a cohort of colorectal cancer patients from Newfoundland (*n* = 505).

## 2. Methods

### 2.1. Ethic Statement

All patients or their family members provided written consent to use of patient biospecimen and access to medical information. This study was approved by the Human Investigation Committee (HIC, recently named as The Health Research Ethics Authority (HREA)) of Memorial University of Newfoundland. During this study, patient-related data were kept anonymous to the researchers who analyzed the data.

### 2.2. Patient Cohort

The patient cohort investigated in this study is a subcohort of the patients recruited to the Newfoundland Colorectal Cancer Registry (NFCCR). NFCCR was described in detail previously [6]. Briefly, in a five-year period (1999–2003), patients diagnosed with colorectal cancer in Newfoundland were contacted and 750 colorectal cancer patients were recruited to NFCCR. Clinical and prognostic data for patients were collected as described in Negandhi et al. [7]. Microsatellite instability (MSI) status for tumor samples was determined using molecular techniques as described by Woods et al. [8]. The last date of follow-up was April 2010. Among 750 patients, 505 Caucasian and unrelated stage I–IV patients for whom the clinical, genetic, and prognostic data were available and whose genotype data passed the quality control measures and satisfied the inclusion criteria were included in this study (see the following paragraph).

Cohort characteristics are summarized in Table 1. The majority of the patients were male (60.8%). Proportion of patients with colon tumors (66%) and MSI-L/MSS tumors (85.4%) were higher than the patients with rectal cancer and MSI-H tumors. The median follow-up times for OS and DFS were 6.36 years (range: 0.38–10.88) and 6 years (range: 0.22–10.88), respectively. During the follow-up, a total of 170 deaths (the number of events for OS analysis) and 200 recurrences, metastases, or deaths (the number of events in the DFS analysis) were observed in this cohort.

### 2.3. Genotyping Experiment, Quality Control Measures, and Inclusion Criteria

The venous blood samples of patients were collected at the time of recruitment and genomic DNA samples were extracted. Genotyping experiments were performed for 539 patients with available prognostic data at a service provider (Centrillion Biosciences, USA) using the Illumina Human Omni1-Quad SNP genotyping platform. This cohort contains >1 million SNP markers. The following quality control (QC) measures were taken to exclude samples: patients with ambiguous gender information (based on the X-chromosome genotype data compared to the self-identified sex, *n* = 1), first, second, and third degree relatives among the patients based on the identity-by-state PHI-hat score (>0.125, *n* = 21), patients with low heterozygosity rate determined based on ±3 SD from the mean (*n* = 1), and non-Caucasian patients based on the multidimensional scaling method and principal component analysis (*n* = 11). Genotyping of no sample was accidently duplicated. All patients had successful genotypes rates of >95%. As a result, 505 unrelated and Caucasian colorectal cancer patients who met these QC measures and inclusion criteria were included in this study (Table 1).

### 2.4. Selected Genes and Polymorphisms

The dbCPCO database [9] summarizes the findings of studies testing the associations of genetic variations with clinical outcomes in colorectal cancer. In brief, genes, polymorphisms, outcomes investigated, patient characteristics, results of the statistical tests for outcome measure(s) investigated, and other relevant information are curated from eligible published reports and are posted in the dbCPCO database. Eligibility criteria for the publications include the following: being a research article and including at least 20 patients in the analysis [9]. For this study, we extracted the genetic polymorphisms that were reported in at least one study/patient cohort to be associated with survival outcomes in multivariable analyses, including overall, disease-free, cancer-specific, and disease-specific survivals, time to recurrence/progression and risks of recurrence or metastasis, and disease progression, as of October 2013. Among these studies, we focused on only the inherited genetic polymorphisms in the form of single nucleotide polymorphisms (SNPs) and insertion/deletion (indels) from the nuclear genes. Microsatellite markers and haplotypes were excluded. In addition, SNPs from genes that are genotyped and investigated by our group in either published [7] (highly similar cohort with many patients in common between the published and the present study cohorts) or still ongoing studies (exactly the same patient cohort; these studies with different research questions/candidate pathway approaches will be published later) were excluded. One SNP with no rs number was also excluded (*RPH3AL* -25C/A in 5′-UTR). As a result, 149 polymorphisms from 94 genes and other loci were initially selected for this project (see Additional File-1 in Supplementary Materials available online at http://dx.doi.org/10.1155/2015/968743).

### 2.5. Selected Genes, Polymorphisms, and Quality Control Measures

**Figure 1:**
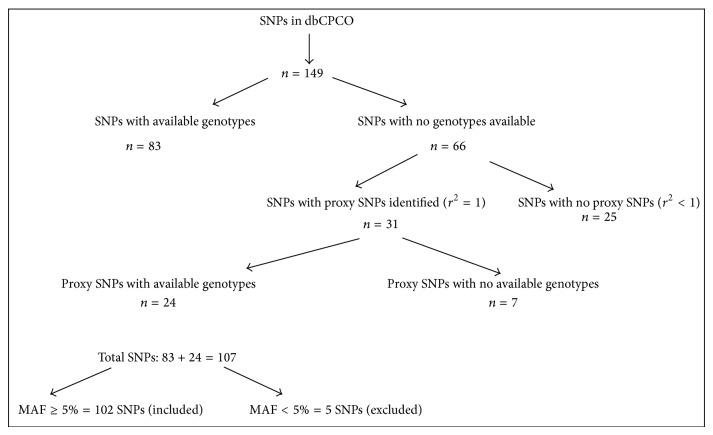
MAF: minor allele frequency; SNP: single nucleotide polymorphism. A flowchart of the process in selecting the SNPs included in this study.

We first searched the availability of genotype data for the polymorphisms listed in Additional File-1 within the genomewide genotype data using PLINK [10]. As a result, genotype data for a total of 83 SNPs were available for our patients. For the remaining SNPs (*n* = 66), we looked for the SNPs that are highly correlated (*r* ^2^ = 1) using the Caucasian (CEU) 1000 genomes Pilot 1 data in the SNAP database (http://www.broadinstitute.org/mpg/snap/ldsearch.php) [11]; we reasoned that these “proxy” SNPs can be used to investigate the associations of these SNPs in our cohort as they have identical genotype distributions (Additional File-1). This search found an at least one proxy SNP for 31 of the 66 polymorphisms. For 24 of such proxy SNPs, there were genotype data available for our patient cohort. Therefore, after these steps, the genotype data of a total of 107 polymorphisms were available in the patient cohort for investigation. The minor allele frequencies (MAFs) of the SNPs in this patient cohort ranged from 2 to 50%. Five SNPs with MAFs less than 5% (rs11208727, rs5789, rs2272990, rs11986055, and rs17226088) were excluded from the statistical analysis. As a result, a total of 102 SNPs were included in the final statistical analysis (Additional File-2). A flowchart of the SNP selection approach is depicted in Figure 1.

Among these SNPs, two SNPs were reported as associated with (at least) DFS, 64 SNPs were reported to be associated with OS, and one SNP was reported to be associated with both OS and DFS by others in the literature; all other studies reported associations of the SNPs with other clinical outcomes; a summary of the previous studies' findings based on the dbCPCO database can be found in Additional File-3.

### 2.6. Statistical Analyses

The outcome measures used in this study were the overall survival (OS), defined as the date from diagnosis till the date of death or the date of last contact (the censored patients) and disease-free survival (DFS) defined as the date from diagnosis till the date of diagnosis of recurrence or metastasis or death, whichever occurred earlier, or the date of last contact.

Initially, genotypes for each SNP were coded under the codominant genetic model, in which the major allele homozygotes, heterozygotes, and minor allele homozygotes were categorized separately and coded as 0, 1, and 2, respectively. Following a previously published approach [12], to estimate the best possible genetic model that fits each polymorphism, Kaplan-Meier survival curves were constructed using the codominant genetic model coding scheme for OS and DFS separately. This step served as a prescreening of SNPs for further statistical analyses and to estimate the best genetic models for promising SNPs based on the pattern of their Kaplan-Meier curves. During this step, SNPs for which a genetic model could not be assigned (either because the SNP genotypes did not show Kaplan-Meier curve separation or the curves were close and/or crossing with each other multiple times) were excluded from further analysis. Of note, for these excluded polymorphisms the log-rank *p* values were supportive of their exclusion (>0.05), except in one case (*HS2ST1*-rs9433110 in overall survival analysis) where the log-rank *p* value was <0.05 due to one patient with the homozygous minor allele genotype who passed away soon after the diagnosis, the curves of two other genotypes (AA and Aa) did not separate; this SNP was therefore excluded from further analysis. While there were other SNPs that had log-rank *p* values > 0.05 in this preselection analysis, they were not excluded because of the separation of their curves that helped us to estimate a suitable genetic model for their analysis. Sometimes more than one genetic model seemed to fit a SNP; in such cases, multiple models were assigned to the SNPs. The SPSS outputs for Kaplan-Meier survival curve analysis for OS and DFS as well as the estimated genetic models are shown in Additional File-4 and Additional File-5, respectively. When the number of patients in the minor allele homozygote group was less than 10, recessive, codominant, and additive models were not attempted; rather the dominant genetic model was applied (if applicable). Once the best genetic model(s) and the SNPs to be excluded were determined, univariate Cox regression analysis was performed for the final list of SNPs under the best genetic models. In the case of SNPs with multiple candidate best genetic models, the one with the smallest *p* value derived from the univariate Cox regression analysis was deemed to be the best genetic model [13]. As per the request of an anonymous reviewer, we also repeated the Cox regression analyses for OS and DFS by adjusting for disease stage.

For SNPs, the reference group changed based on the genetic model. For example, assuming AA, Aa, and aa donate major allele homozygote, heterozygote, and minor allele homozygote genotypes, the reference groups in recessive, dominant, and codominant genetic models were AA+Aa, AA, and AA, respectively.

To control for the false-positive rate, the False Discovery Rate (FDR) method [14] was used; the cutoff of the FDR adjusted *p* value (*q* value) was 0.05. In this paper, polymorphisms that showed no statistically significant associations after FDR adjustment (adjusted *p* < 0.05) but had an unadjusted *p* < 0.01 are noted as nominally associated with the outcomes.

We computed the power of the samples for genetic association tests. Given a SNP in LD (*D*′ = 1) with a risk allele frequency 0.3, we have 85% power to detect a significant association at nominal significance *p* value < 0.01 under a dominant model with strong effect size of HR 1.8. To detect an association with the same assumptions and FDR adjusted *p* value < 0.05, the statistical power is reduced to 0.61. With a moderate effect size of HR 1.5, the power to detect significant association (FDR adjusted *p* < 0.05) is very low.

Testing the proportionality assumption for Cox univariate model was done based on the Schoenfeld residual analysis using the “survival” package of R software [15]. The cox.zph function calculates tests of the proportional hazards assumption for each covariate. *p* value greater than 0.05 means the proportional hazard assumption is valid. FDR calculations were done using the R function p.adjust. Other statistical analyses were performed by the SPSS (version 20, IBM, USA) or R [15]. PLINK [10] was used for genotype quality control and summary purposes.

### 2.7. PolyPhen-2 Analysis

A possible biological effect of the nonsynonymous rs197412 polymorphism (Ile636Thr) on DDX20 protein function was examined using the PolyPhen-2 tool [16].

## 3. Results and Discussion

In this study, we aimed to test the previously reported associations of 102 genetic polymorphisms with survival outcomes of colorectal cancer patients in a separate patient cohort using overall and disease-free survivals as the outcome measures. While the previous associations were detected with a variety of outcomes (including overall, progression-free, recurrence-free, cancer-specific or disease-specific survivals, time-to-recurrence and others), we reasoned that they may be good candidates to systematically investigate in relation to overall and disease-free survivals (two outcome measures available for our patient cohort).

Genotype distributions of none of the 102 SNPs deviated from the HWE (*p* values > 0.01). Genes, SNPs and their polymorphic alleles, minor allele frequencies, the best genetic models estimated for OS and DFS using the Kaplan-Meier curves, and the best genetic models selected by the univariate Cox regression method (in the case of SNPs with more than one candidate genetic models) are shown in Additional File-2.

For overall survival, at least one genetic model was assigned to 65 of the 102 SNPs. For DFS, at least one genetic model was estimated for 78 SNPs. These SNPs were further investigated by statistical analyses (please see the following paragraph). In both overall and disease-free survival analyses, the recessive genetic model was the most frequently chosen model while the additive model was the least chosen one.

After the genetic model estimations, the univariate Cox regression analysis was performed for the 65 SNPs in overall survival and 78 SNPs in disease-free survival analysis under the genetic models selected for them (Additional File-6 and Additional File-7). Initially, our univariate analysis results suggested nominal associations of four polymorphisms (unadjusted *p* < 0.01). Specifically, the minor allele containing genotypes (genotypes AG and GG) for the* IFNGR1*-rs1327474 polymorphism were associated with longer overall survival times when compared to the major allele homozygous genotype (AA) (Table 2).* IFNGR1*-rs1327474 polymorphism (NM_000416.2:c.-611G>A) is located near the 5′-end of the* IFNGR1* gene [17] that codes for the ligand-binding chain of the interferon gamma receptor 1 [18]. Previously association of this polymorphism with disease-specific survival in colorectal cancer was reported [19]. In addition, patients heterozygous for the* RRM1*-rs12806698 polymorphism had shorter overall survival times compared to patients with the major allele homozygous (CC) genotype (Table 2). According to the dbSNP database [17], the* RRM1*-rs12806698 (NM_001033.3:c.-269C>A; also called -37A/C) is a C/A substitution located in the 5′-UTR of the* RRM1* gene possibly affecting its expression [20]. A study previously reported its association with time-to-progression in colorectal cancer patients treated with gemcitabine [20].

In univariate disease-free survival analysis, nominal associations (unadjusted *p* values of <0.01) were detected for three polymorphisms (Table 2). Similar to overall survival results, patients with the AG and GG genotypes of the* IFNGR1*-rs1327474 polymorphism had longer disease-free survival times compared to patients with the AA genotype. Additionally, each C allele increased the disease-free survival times in patients in the case of* DDX20*-rs197412 polymorphism. DDX20 (also called GEMIN3) is presumably a RNA helicase involved in RNA processing [21].* DDX20*-rs197412 polymorphism results in a nonsynonymous amino acid substitution (NP_009135.4:p.Ile636Thr T/C [17]). Based on our analysis using the PolyPhen-2 tool [16], this nonsynonymous polymorphism is not predicted to affect the protein function. Two other studies have so far investigated this polymorphism in relation to survival outcomes in colorectal cancer; while one study [22] found it associated with recurrence-free survival, another study did not associate it with overall and progression-free survivals [23]. Finally, patients heterozygous for the* PTGS2*-rs5275 polymorphism had worse disease-free survival times compared to patients homozygous for the major allele (TT genotype).* PTGS2* (also known as Cox-2) encodes for cyclooxygenase enzyme that functions in inflammation-related pathways [24]. The rs5275 polymorphism (NM_000963.2:c.∗427T>C) is located in the 3′-UTR of the* PTGS2* gene [17]. This polymorphism was previously predicted to be in a miRNA binding sequence; however experimental analyses in lymphoblastoid cell lines did not find an association of it with the* PTGS2* expression levels [25]. In a previous study, this polymorphism was found to be associated with progression-free and overall survivals in colorectal cancer [26].

When the SNP genotypes were adjusted for disease stage, no associations of the* IFNGR1*-rs1327474 and* RRM1*-rs12806698 with OS were detected (*p* values > 0.01). However, in DFS analysis, the nominal associations (*p* values < 0.01) were obtained for the* DDX20*-rs197412 and the* PTGS2*-rs5275 genotypes (Table 2). Additionally, patients with the minor allele containing genotypes (TC and TT) of the* HSPA5*-rs391957 polymorphism had longer disease-free survivals when compared to patients with the major allele homozygous genotype (CC) (Table 2). HSPA5 is a heat-shock protein involved in protein folding activity within the endoplasmic reticulum [27]. The C allele of this polymorphism was previously shown to create a transcription factor binding site and to be associated with increased expression of the* HSPA5 *gene [28]. In colorectal cancer, this polymorphism was previously associated with time-to-recurrence in a patient cohort [29].

**Figure 2:**
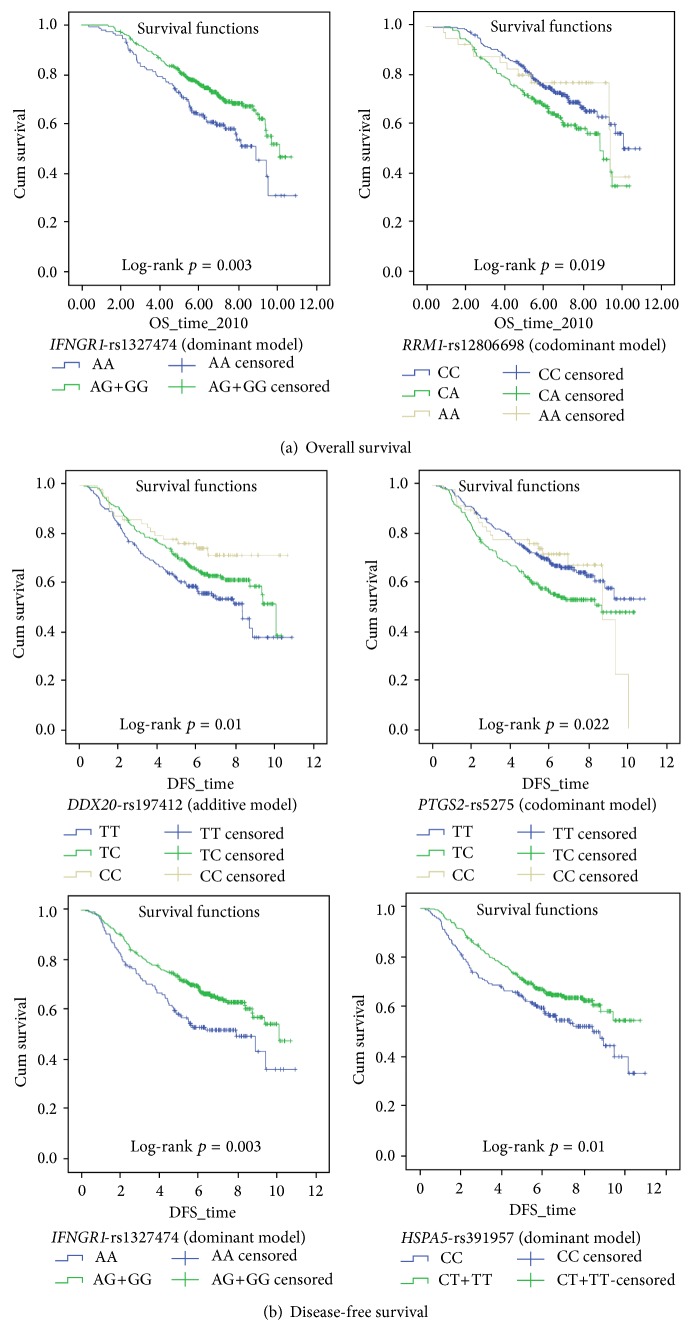
The *p* values shown are obtained by the log-rank test. DFS: disease-free survival; OS: overall survival. Kaplan-Meier survival curves for the polymorphisms with nominal associations (*p* value < 0.01).

Kaplan-Meier curves for SNPs discussed are shown in Figure 2. Of note, none of the five SNPs violated the Cox proportional hazards assumption.

We then applied the FDR method to account for multiple comparisons. After FDR correction, association of none of the polymorphisms with outcomes remained significant. Therefore, we can conclude that none of the investigated polymorphisms showed statistically significant associations with overall survival and disease-free survival in our patient cohort. However, this may be in part because of the limited sample size and the insufficient study power. Therefore, a large cohort with a longer follow-up time may be needed for further assessment of these genetic markers.

Limitations of this study should be mentioned as follows. (i) While the polymorphisms were selected based on their associations with a variety of survival outcomes in previous studies on colorectal cancer, our study was limited with testing their associations with overall survival and disease-free survival only. In addition, the cohorts might have differed in terms of ethnicity or other population and disease related characteristics, and the genetic models used during the statistical analyses are likely to be different, too. Due to these differences between our study and other studies, the results of this study and other studies hence should be compared with caution. (ii) Not all polymorphisms first identified through the literature could be studied in this study due the lack of their genetic data in our cohort. (iii) When compared to the entire NFCCR patient cohort, the study cohort had a higher proportion of stage I–III patients: this was due to the low number of stage IV patients with DNA samples that was available for the genotyping experiment.

## 4. Conclusions

In conclusion, there was no evidence of associations of the 102 polymorphisms investigated in this study with overall or disease-free survivals in a colorectal cancer patient cohort from Newfoundland.

## Supplementary Material

Additional information and data related to this study is summarized in the Supplementary Material Excel document. Additional File 1: Information on the initial 149 SNPs. Additional File 2: SNPs (n=102) included in this study, allele frequencies, and genetic models. Additional File 3: Summary of previous literature reports on SNPs. Additional File 4: Kaplan-Meier curves (overall survival analysis). Additional File 5: Kaplan-Meier curves (disease free survival analysis). Additional File 6: Univariate and stage-adjusted Cox regression analysis results (overall survival). Additional File 7: Univariate and stage-adjusted Cox regression analysis results (disease-free survival).

## Figures and Tables

**Table 1:** Clinicopathological, molecular, and treatment-related characteristics of the patients included in this study.

Variables	*n* (%)
Sex	
Female	198 (39.21)
Male	307 (60.79)
Age at diagnosis	Median: 61.43 years (range: 20.7–75)
Histology	
Nonmucinous	448 (88.71)
Mucinous	57 (11.29)
Location	
Colon	334 (66.14)
Rectum	171 (33.86)
Stage	
I	93 (18.42)
II	196 (38.81)
III	166 (32.87)
IV	50 (9.9)
Grade	
Well/moderately differentiated	464 (91.88)
Poorly differentiated	37 (7.33)
Unknown	4 (0.79)
Vascular invasion	
Absent	308 (60.99)
Present	159 (31.49)
Unknown	38 (7.52)
MSI status	
MSI-L/MSS	431 (85.35)
MSI-H	53 (10.5)
Unknown	21 (4.16)

**Table 2:** Results of the univariate and multivariable Cox regression analysis for the SNPs that are nominally associated with overall or disease-free survivals (*p* < 0.01).

Genetic model	*n*	SNP	*p* value	HR	95% CI for HR		*q* value	*p* value	HR	95% CI for HR		*q* value
					Lower	Upper				Lower	Upper	
			OS (univariate)					OS (adjusted for stage)				
Dominant	504	*IFNGR1*_rs1327474 (AG+GG vs AA)	**0.003**	0.624	0.457	0.853	0.243	0.022	0.691	0.504	0.947	0.351
		*RRM1*_rs12806698	0.02				0.389	0.045				0.456
Codominant	504	*RRM1*_rs12806698 (CA vs CC)	**0.008**	1.524	1.116	2.082	0.324	0.015	1.472	1.077	2.011	0.351
		*RRM1*_rs12806698 (AA vs CC)	0.833	0.934	0.497	1.757	0.865	0.941	1.024	0.542	1.935	1

			DFS (univariate)					DFS (adjusted for stage)				
Additive	503	*DDX20*_rs197412	**0.003**	0.714	0.574	0.889	0.159	**0.004**	0.725	0.581	0.905	0.212
Dominant	503	*IFNGR1*_rs1327474 (AG+GG vs AA)	**0.003**	0.642	0.480	0.859	0.159	0.017	0.701	0.524	0.939	0.3
Dominant	503	*HSPA5*_rs391957 (TC+TT vs CC)	0.011	0.692	0.521	0.918	0.233	**0.007**	0.676	0.509	0.897	0.247
		*PTGS2*_rs5275	0.023				0.327	0.011				0.254
Codominant	503	*PTGS2*_rs5275 (TC vs TT)	**0.009**	1.477	1.100	1.984	0.233	**0.004**	1.541	1.147	2.071	0.212
		*PTGS2*_rs5275 (CC vs TT)	0.974	1.008	0.617	1.646	0.974	0.872	1.041	0.637	1.702	0.919

CI: confidence interval; DFS: disease-free survival; HR: hazards ratio; *n*: number of patients in the analysis; OS: overall survival; SNP: single nucleotide polymorphism.
